# Perioperative screening and management of hyperglycemia: a joint position statement from the Brazilian Diabetes Society (SBD), the Brazilian Society of Anesthesiology (SBA) and the Brazilian Association for the Study of Obesity and Metabolic Syndrome (ABESO)

**DOI:** 10.1186/s13098-025-02060-5

**Published:** 2026-02-27

**Authors:** Emerson Cestari Marino, Leandra Anália Freitas Negretto, Rogério Silicani Ribeiro, Denise Momesso, Alina Coutinho Rodrigues Feitosa, Marcos Tadashi Kakitani Toyoshima, Joaquim Custódio da Silva Junior, Sérgio Vencio, Marcio Weissheimer Lauria, João Roberto de Sá, Domingos A. Malerbi, Fernando Valente, Silmara A. O. Leite, Danillo Ewerton Oliveira Amaral, Gabriel Magalhães Nunes Guimarães, Plínio da Cunha Leal, Maristela Bueno Lopes, Luiz Carlos Bastos Salles, Liana Maria Torres de Araújo Azi, Amanda Gomes Fonseca, Lorena Ibiapina M. Carvalho, Francília Faloni Coelho, Bruno Halpern, Cynthia M. Valerio, Fabio R. Trujilho, Antonio Carlos Aguiar Brandão, Ruy Lyra, Marcello Bertoluci

**Affiliations:** 1 Curitiba Diabetes Center, Curitiba, Brazil; 2 Endocrinology Service, Hospital Nossa Senhora das Graças, Curitiba, Brazil; 3 Hospital Israelita Albert Einstein, Goiânia, Brazil; 4 Hospital das Clínicas da Universidade Federal de Goiás, Goiânia, Brazil; 5 Hospital Israelita Albert Einstein, São Paulo, Brazil; 6 Agile Healthtech, São Paulo, Brazil; 7 Endocrinology Service, Universidade Federal do Estado do Rio de Janeiro, Rio de Janeiro, Brazil; 8 Clínica São Vicente, Rede D’Or- São Luiz, Rio de Janeiro, Brazil; 9 Endocrinology Service, Hospital Santa Izabel da Santa Casa da Bahia, Salvador, Brazil; 10 Endocrine Service, Maternidade Maria Conceição de Jesus, Salvador, Brazil; 11 Endocrine Oncology Unit, Instituto do Câncer do Estado de São Paulo Octavio Frias de Oliveira, Hospital das Clínicas da Faculdade de Medicina da Universidade de São Paulo, São Paulo, Brazil; 12 Laboratório de Carboidratos e Radioimunoensaio LIM/18, Divisão de Endocrinologia e Metabologia, Hospital das Clinicas, Faculdade de Medicina da Universidade de Sao Paulo, São Paulo, Brazil; 13 Faculdade de Medicina da Bahia, Universidade Federal da Bahia, Salvador, Brazil; 14 Complexo Hospitalar Universitário Professor Edgard Santos, Universidade Federal da Bahia, Salvador, Brazil; 15 Universidade Evangélica de Goias, Goiânia, Brazil; 16 Hospital das Clínicas da Universidade Federal de Minas Gerais, Belo Horizonte, Brazil; 17 Hospital Felicio Rocho, Belo Horizonte, Brazil; 18 Disciplina de Endocrinologia, Centro Universitário Faculdade de Medicina Do ABC, Santo André, Brazil; 19 Disciplina de Endocrinologia, Escola Paulista de Medicina - UNIFESP/EPM, São Paulo, Brazil; 20 Hospital Israelita Albert Einstein, São Paulo, Brazil; 21 Sociedade Brasileira de Diabetes (SBD), São Paulo, Brazil; 22 Cline Research Center-Curitiba, Paraná, Brazil; 23 Anesthesiology Service, Santa Casa de Misericórdia de Maceió, Maceió, Brazil; 24 Clínica de Anestesiologia de Maceió - CAM, Maceió, Brazil; 25 Hospital Sirio Libanês, Brasília, Brazil; 26 Sociedade Brasileira de Anestesiologia, Rio de Janeiro, Brazil; 27 Programa de Pós-Graduação em Saúde do Adulto, Universidade Federal do Maranhão, São Luís, Brazil; 28 Programa de Pós-Graduação em Ciência Cirúrgica Interdisciplinar, Universidade Federal de São Paulo, São Paulo, Brazil; 29 Hospital São Marcelino Champagnat – Pontifícia Universidade Católica do Paraná (PUC-PR), Curitiba, Brazil; 30 Universidade Federal do Estado do Rio de Janeiro, Rio de Janeiro, Rio de Janeiro, Brazil; 31 Universidade Federal da Bahia, Salvador, Brazil; 32 Hospital e Maternidade Marieta Konder Bornhousen, Itajaí, Brazil; 33 Hospital Unimed Primavera, Teresina, Brazil; 34 Américas Medical City, Rio de Janeiro, Brazil; 35 Grupo de Obesidade, Departamento de Endocrinologia, Hospital das Clínicas da Universidade de São Paulo, São Paulo, Brazil; 36 State Institute of Diabetes and Endocrinology Luiz Capriglione (IEDE-RJ), Department of Metabolism, Rio de Janeiro, Brazil; 37 Coordinator of the Weight Loss Maintenance Department, Institutional Consultant and Researcher at the Obesity Hospital, Salvador, Brazil; 38 Centro de Diabetes e Endocrinologia do Estado da Bahia ( CEDEBA), Salvador, Brazil; 39 Núcleo de Pesquisa Do Hospital da Obesidade, Salvador, Brazil; 40 Hospital das Clínicas Samuel Libânio, Pouso Alegre, Brazil; 41 Universida de Federal de Pernambuco, Recife, Brazil; 42 Faculdade de Medicina da Universidade Federal do Rio Grande do Sul, Porto Alegre, Rio Grande do Sul Brazil; 43 Serviço de Endocrinologia, Hospital de Clínicas de Porto Alegre, Porto Alegre, Rio Grande do Sul Brazil

## Abstract

**Background:**

Hospital hyperglycemia, whether or not diabetes has been previously diagnosed, increases the risk of postoperative complications, particularly infections, prolonged hospital stay, and in-hospital mortality. Ensuring adequate glycemic control during the perioperative period helps reduce these risks and improve surgical outcomes.

**Methods:**

This guideline was developed by the Department of Acute Complications and Inpatient Glycemic Control of the Brazilian Diabetes Society (SBD), in collaboration with representatives from the Brazilian Society of Anesthesiology (SBA), the Brazilian Association for the Study of Obesity and Metabolic Syndrome (ABESO), and the SBD Scientific Committee. Key clinical questions guided a narrative review of the literature using MEDLINE via PubMed, including evidence from randomized clinical trials (RCTs), meta-analyses, and high-quality observational studies.

**Results and conclusions:**

The expert group produced 19 recommendations and 20 key notes addressing screening, glycemic targets, management of oral and injectable agents, insulin strategies, and prevention of perioperative complications. Based on the best available evidence, this joint statement provides practical and effective guidance for managing perioperative hyperglycemia across different levels of healthcare.

## Introduction

Hospital hyperglycemia, regardless of prior diagnosis of diabetes mellitus, is associated with an increased risk of complications in surgical patients. Individuals with hyperglycemia tend to experience longer hospital stays, a higher incidence of nosocomial infections, particularly surgical site infections, and increased in-hospital mortality. These risks are especially pronounced among patients without a prior diagnosis of diabetes [1–3].

Proper perioperative glycemic control has been associated with reduced rates of these complications and improved clinical outcomes during hospitalization (Table 1) [4–7].

**Table 1:** Goals of preoperative blood glucose assessment and diabetes management [1–3, 5, 7]

● Detect previously undiagnosed cases of diabetes mellitus; ● Optimize glycemic control in patients with known but poorly controlled diabetes; ● Assess the risk of perioperative complications related to hyperglycemia; ● Adjust antidiabetic medications and insulin dosing in the surgical setting; ● Maintain adequate glycemic control and ensure appropriate perioperative nutritional intake; ● Prevent postoperative complications; ● Plan the safe and effective transition of care from hospital to outpatient follow-up	

For outpatients or for patients with an expected hospital stay of less than 48 h, the recommendations from the chapter *“Management of antidiabetic therapy in T2DM”* [8] should be followed. Particular attention should be given to decisions regarding whether to continue or withhold medications, as outlined below.

In contrast, for patients with a projected hospital stay longer than 48 h, those undergoing emergency surgery, or those presenting with active infections, it is recommended to follow the specific protocols for critically and non-critically ill patients, as established by the guidelines of the Brazilian Diabetes Society (SBD). [9]

This review brought together the three societies to align recommended practices across specialties, thereby strengthening communication among patients, surgical teams, clinicians, endocrinologists, and anesthesiologists. It is intended for all professionals involved in perioperative care and represents the first jointly developed Brazilian guideline on this topic.

## Methodology

This review updates the 2025 Brazilian Diabetes Society (SBD) guideline. A steering committee approved the methodology, identified key clinical questions, and conducted a narrative review using MEDLINE.

In brief, the SBD appointed a central committee of experts responsible for overseeing the methodological process, reviewing the manuscript, and assigning the level of evidence and strength of recommendations. The Department of Acute Complications and Inpatient Glycemic Control of the SBD coordinated the effort in collaboration with members of the SBD Scientific Committee and representatives from the Brazilian Society of Anesthesiology (SBA) and the Brazilian Association for the Study of Obesity and Metabolic Syndrome (ABESO).

Key clinical questions were identified, and a narrative review was conducted using the MEDLINE database via PubMed. The best available evidence was considered, including randomized clinical trials (RCTs), meta-analyses, and high-quality observational studies related to perioperative glucose control.

## Level of evidence

Three levels of evidence were considered:

- A—Data from more than one RCT or from meta-analyses of RCTs with low heterogeneity (I^2^< 25%).
- B—Data from meta-analyses with high heterogeneity (I^2^≥ 25%), a single RCT, prespecified subgroup analysis, large observational studies, or meta-analyses of observational studies.
- C—Data from small or nonrandomized studies, exploratory analyses, clinical practice guidelines, or expert consensus statements.

## Degree of recommendation

Each recommendation was submitted for voting by members of the Department of Acute Complications and Inpatient Glycemic Control and the central committee. The level of agreement determined the strength of the recommendation, as follows:

- I—IS RECOMMENDED: > 90% agreement among panel members;
- IIa—SHOULD BE CONSIDERED: 70–90% agreement;
- IIb—MAY BE CONSIDERED: 50–70% agreement; and
- III—IS NOT RECOMMENDED: < 50% agreement or majority against.

## Recommendations


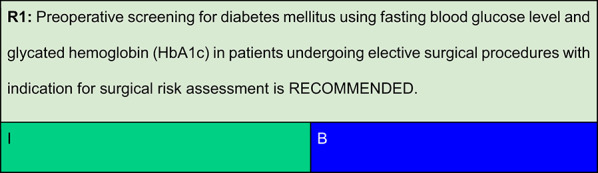


## Summary of evidence

- Abdelmalak et al. reported that approximately 10% of patients undergoing non-cardiac surgery had previously undiagnosed diabetes. These individuals presented with significantly higher preoperative glucose levels than those with known diagnosis. [10]
- Similarly, Lauruschkat et al. found that undiagnosed diabetes in patients undergoing cardiac surgery was associated with worse postoperative outcomes, including higher rates of reintubation, prolonged mechanical ventilation, and increased in-hospital mortality. [11]
- Panayi et al., analyzing 502,478 individuals in the American College of Surgeons National Surgical Quality Improvement Program database (ACS-NSQIP, 2021–2022) with recorded HbA1c results, identified a 5% prevalence of undiagnosed diabetes. This condition was independently associated with increased postoperative complications (8.9%). [12]
- In Brazil, a significant proportion of individuals with diabetes remain undiagnosed. Therefore, screening with fasting plasma glucose and HbA1c is recommended for patients without a known diagnosis, especially those aged ≥ 35 years or with the following risk factors for type 2 diabetes, including: [13]
  - First-degree family history of diabetes;
  - History of cardiovascular disease;
  - Hypertension;
  - HDL-cholesterol < 35 mg/dL;
  - Triglycerides > 250 mg/dL;
  - Polycystic ovary syndrome;
  - Acanthosis nigricans;
  - Sedentary lifestyle;
  - Prior history of prediabetes;
  - History of gestational diabetes or macrosomic birth;
  - High or very high FINDRISC score;
  - Classic symptoms of hyperglycemia.
- Miller et al. emphasized that the preoperative assessment represents an important opportunity to detect previously unrecognized diabetes and optimize glycemic control in patients with known diabetes, including screening for chronic complications. [14]

**Table:** 

Important note 1: Glycated hemoglobin (HbA1c)	
● HbA1c testing is useful both for diagnosing diabetes and for evaluating prior glycemic control. It should not delay surgical clearance but does help stratify perioperative risk. Abnormal HbA1c values indicate a higher likelihood of hospital hyperglycemia and justify closer monitoring during the perioperative period. [13] ● In patients without a prior diabetes diagnosis, HbA1c levels between 5.7% and 6.4% warrant a 75 g oral glucose tolerance test (OGTT), with plasma glucose measured 1 or 2 h post-ingestion, as recommended by the Brazilian Diabetes Society guideline for the diagnosis of diabetes mellitus ● HbA1c may be measured either before or after the preoperative evaluation; however, abnormal values should prompt individualized perioperative planning and more vigilant glucose monitoring. [13, 15]	


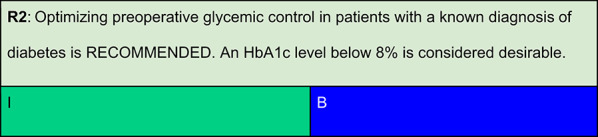


## Summary of evidence

Association between HbA1c and postoperative mortality.

Van den Boom et al., analyzing 431,480 surgical procedures from the Duke University Health System, demonstrated a strong association between preoperative HbA1c, mean postoperative capillary glucose during the first 3 days, and 30-day mortality. These findings reinforce the prognostic value of glycemic control in the perioperative setting [2].

Impact of HbA1c on cardiac surgery outcomes.

- Halkos et al., in a prospective cohort of 3,089 patients undergoing elective coronary artery bypass grafting (CABG), reported that higher preoperative HbA1c values were associated with increased risks of in-hospital mortality (odds ratio [OR] = 1.40; p = 0.02), myocardial infarction (OR = 1.55; p = 0.05), and deep sternal wound infection (OR = 1.38; p = 0.03). Notably, HbA1c level > 8.6% was linked to a four-fold increase in mortality. [16]
- By contrast, Tsuruta et al., studying 306 Japanese patients with diabetes undergoing cardiac procedures, did not find a significant association between HbA1c and long-term mortality or complications such as mediastinitis, acute renal failure, or arrhythmias. Patients were stratified into < 6.5%, 6.5–7.5%, and ≥ 7.5% groups, and outcomes were similar across categories. [17]
- Subramaniam et al. showed that HbA1c ≥ 6.5% predicted a higher incidence of adverse in-hospital outcomes (including in-hospital mortality, acute myocardial infarction, reoperation, sternal wound infection, cardiac tamponade, pneumonia, stroke, and renal failure) in 1,461 patients undergoing CABG (OR = 1.5; 95% CI, 1.1–2.3; p = 0.02). [18]
- In a Swedish cohort of 764 patients with type 1 diabetes (T1DM) undergoing elective CABG, Nyström et al. found that HbA1c > 10.0% more than doubled the risk of major adverse cardiovascular events compared to HbA1c ≤ 7.0% (HR = 2.25; 95% CI, 1.29–3.94). Each 1% increase in HbA1c raised risk by 18% (HR = 1.18; 95% CI, 1.06–1.32). After a median follow-up of 4.7 years, 44% of participants died or had major adverse cardiovascular events (MACE: myocardial infarction, stroke, heart failure, or repeat revascularization). [19]

Impact of HbA1c on non-cardiac surgery.

- Underwood et al. reported that HbA1c > 8% was associated with longer hospital stays in patients undergoing non-cardiac surgeries at Brigham and Women’s Hospital between 2005 and 2010. They suggested that improving glycemic control prior to surgery may enhance postoperative outcomes. [20]
- Seisa et al., in a systematic review of 44 nonrandomized studies involving 127,791 patients, found that HbA1c < 7% was associated with shorter length of stay (-0.5 days), lower postoperative glucose levels (-46.5 mg/dL), and reduced incidence of infections (-46%) and neurologic (-49%) complications. However, there was also a higher rate of reoperations (+ 40%), and the quality of evidence was rated as low or very low. [21]

HbA1c thresholds and postponement of surgery.

- Sethuraman et al. noted that there is no consensus regarding the optimal HbA1c threshold for safe surgery. While HbA1c ≤ 7% may be preferred for high-risk procedures (e.g., spine, joints, cardiac), levels up to 8% may be acceptable for most surgeries. [22]
- Panayi et al. reported a nonlinear association between HbA1c and postoperative complications in the ACS-NSQIP cohort. The complication rates were 9.5% for HbA1c 6.0–6.9% and increased to 14.5% for HbA1c > 9%. The lowest risk was observed among patients with HbA1c between 7 and 8%. [12]
- Simha et al. recommend postponing elective procedures when HbA1c > 8%, when feasible, to allow optimization of glycemic control. In cases of severe hyperglycemia (glucose > 250 mg/dL), they advise delaying surgery regardless of the presence of metabolic decompensation [1].

Clinical perspective

While evidence-based thresholds help guide decision-making, perioperative glycemic management must always be individualized. Elderly patients and those with multiple comorbidities may be particularly vulnerable to hypoglycemia, which may pose greater harm than moderate hyperglycemia. Clinical judgment remains essential to balance safety, feasibility, and metabolic stability for each patient.While evidence-based thresholds help guide decision-making, perioperative glycemic management must always be individualized. Elderly patients and those with multiple comorbidities may be particularly vulnerable to hypoglycemia, which may pose greater harm than moderate hyperglycemia. Clinical judgment remains essential to balance safety, feasibility, and metabolic stability for each patient.

**Table:** 

Important note 2: When HbA1c is out of the target range	
● For patients with a known diabetes, referral to an endocrinologist is recommended when: ○ HbA1c > 8%, indicating suboptimal control, or ○ HbA1c < 6% in patients treated with insulin or insulin secretagogues (sulfonylureas and meglitinides/glinides), due to increased risk of hypoglycemia ● In these situations, elective procedures may be postponed to allow reassessment of glycemic patterns and therapeutic adjustment, reducing perioperative risk ● For time-sensitive procedures—those ideally performed within 1–6 weeks (e.g., oncologic surgeries, neurosurgical decompression, coronary bypass, gynecologic hemorrhage, or limb-threatening fractures)— endocrinology evaluation is strongly advised even HbA1c > 8%. [1]	

**Table:** 

Important note 3: Surgical timing and assessment of ketonemia in elective surgical procedures	
● Elective procedures should ideally be scheduled early in the day to minimize fasting duration ● Blood glucose should be measured on the day of surgery. If levels are > 250 mg/dL, consider postponing the surgical procedure until adequate glycemic control is achieved. Adjustments may be made up to 4 h before the scheduled procedure time ● In patients with type 1 diabetes (T1DM) or those using basal insulin, capillary ketone testing (preferably capillary β-hydroxybutyrate) is recommended in the following scenarios: [23, 24] ○ Presence of metabolic acidosis on blood gas analysis; ○ Clinical suspicion of diabetic ketoacidosis (DKA), such as nausea, vomiting, tachypnea, dehydration, or abdominal pain; ○ Omission of any dose of basal insulin dose; ○ Use of sodium-glucose cotransporter-2 inhibitors (SGLT2i) ■ Note: DKA may occur with only mild hyperglycemia or even normoglycemia, especially in patients using SGLT2i, in those with reduced oral intake, during pregnancy, or with renal impairment. This condition is known as euglycemic diabetic ketoacidosis (EDKA) and can also affect individuals who do not use insulin in high-risk scenarios ● Reference values for capillary ketonemia: [23, 24] ○ Normal: < 0.6 mmol/L ○ Probable DKA: > 3 mmol/L ○ Intermediate values suggest increasing risk and require immediate intervention with hydration, insulin infusion, and, if needed, caloric and potassium supplementation (see corresponding chapters on DKA and EDKA)	

**Table:** 

Important note 4: Non-elective surgical procedures	
● For urgent or emergency procedures—when postponement is not feasible—early involvement of an endocrinologist is recommended to ensure appropriate acute glycemic management ● In emergency situations, where it is not possible to assess clinical symptoms (as described in Important Note 3), and information about diabetes or insulin use is unavailable, capillary ketonemia testing is advisable. If capillary ketones are not available, urine ketone testing should be performed in patients with blood glucose levels > 200 mg/dL to help identify and promptly treat diabetic ketoacidosis (DKA). [23, 24] ● In cases of severe hyperglycemia (typically > 600 mg/dL), the possibility of hyperosmolar hyperglycemic state (HHS) should be considered, and immediate treatment initiated due to its high morbidity and mortality	


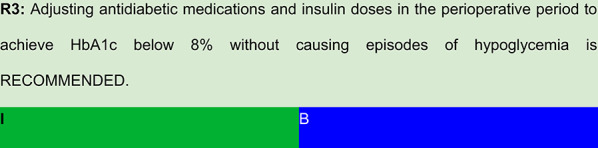


## Summary of evidence

- Multiple reviews, clinical studies, and retrospective analyses have consistently demonstrated that perioperative glycemic control significantly influences surgical outcomes. Failure to adjust antidiabetic therapy appropriately is linked to higher rates of postoperative complications. Accordingly, tailoring antidiabetic treatment—whether oral medications, non-insulin injectables, or insulin—is essential to ensure safe and effective glycemic targets during the perioperative period. [2, 20, 22, 25, 26]
- Specific recommendations on continuation or temporary discontinuation of individual drug classes are discussed in detail in the subsequent sections of this guideline.

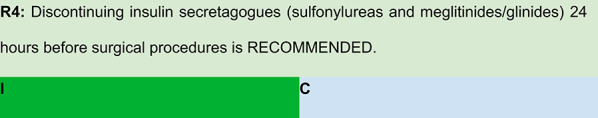


## Summary of evidence

- A literature review identified a high risk of hypoglycemia in patients using insulin secretagogues—such as sulfonylureas and meglitinides—with up to 70% of individuals with diabetes experiencing at least one hypoglycemic episode while taking these medications. Because they stimulate insulin release regardless of food intake, these agents pose a particular risk during perioperative fasting. [25]
- Although rarely prescribed today, chlorpropamide has a particularly long half-life. For patients who are still taking this medication, discontinuation 48–72 h before surgery is recommended. Such cases warrant preoperative evaluation by an endocrinologist to determine the safest alternative treatment strategy.

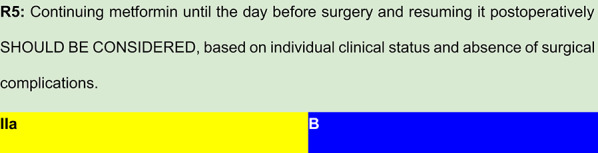


## Summary of evidence

- Bano et al. analyzed a cohort of 1,800 patients undergoing coronary artery bypass grafting (CABG) to assess the safety of continuing metformin until the night before surgery. In the final analysis, postoperative lactate levels were similar across patients who continued metformin, patients with diabetes not using metformin, and those without diabetes. [27]
- A Cochrane meta-analysis including 347 prospective and cohort studies (70,490 patient-years in the metformin group and 55,451 patient-years in the non‐metformin group) found no significant difference in the incidence of lactic acidosis between metformin users (4.3 cases per 100,000 patient-years) and non-users (5.4 per 100,000). However, these data do not specifically refer to perioperative use. [28]
- Nazer et al., in a case–control study, also found no association between continued metformin use before CABG and lactic acidosis. Interestingly, non-metformin users had higher mean peak lactate levels. [29]
- In a randomized controlled trial of patients with T2DM undergoing non-cardiac surgery, Hulst et al. found that perioperative continuation of metformin did not increase lactate levels to clinically meaningful levels, even during preoperative fasting. [30]

## Clinical interpretation

Collectively, these findings suggest that metformin can be safely continued until the day before surgery in most patients. However, its postoperative reintroduction should be guided by the patient’s hemodynamic stability, renal function, and the absence of surgical complications that could predispose to tissue hypoxia or renal impairment.
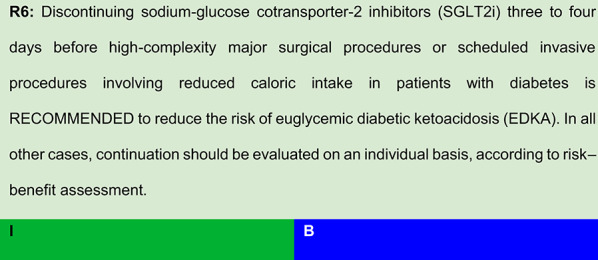


## Summary of evidence

### Risk of EDKA and impact of SGLT2i discontinuation

In a retrospective analysis of 36,505 admissions, Singh et al. compared patients with T2DM who continued SGLT2i during hospitalization (n = 5,936) with those who discontinued them (n = 30,569). After adjustment for confounders (severity, age, sex, body mass index, ethnicity, insulin use, and surgical procedures), continuation was associated with 45% lower in-hospital mortality (RR = 0.55; 95% CI, 0.42–0.73; p < 0.01), without an increase in acute kidney injury, and with a modest reduction in length of stay [31]. However, perioperative use may increase the risk of EDKA, a potentially life-threatening complication [32].

## Role of dietary restriction in EDKA

Thiruvenkatarajan et al., in a systematic review, identified reduced carbohydrate intake as a major contributor to perioperative EDKA. They recommend close monitoring and ketone testing whenever suggestive symptoms occur, particularly when caloric intake is reduced, even if glucose levels are normal [33].

## Preoperative discontinuation of SGLT2i and incidence of EDKA

- A multicenter retrospective cohort including 1,307 patients reported eight cases of EDKA, five occurring during emergency surgery. In contrast, when SGLT2i were discontinued at least three days before elective surgery, the incidence of EDKA was very low. [34]
- Seki et al., in a systematic review of 99 published cases of perioperative ketoacidosis associated with SGLT2i use, found no episodes when the medication was discontinued at least 3 days before surgery. The main risk factors included prolonged fasting, surgical stress, and fluid mismanagement. Bariatric and cardiac surgeries were the most commonly involved. The findings support the 2020 FDA´s guidance, which recommends discontinuing 3–4 days before surgical procedures, although further research is still needed. [35]

## Severity and resolution of DKA associated with SGLT2i

Umapathysivam et al., in a retrospective cohort study, found that SGLT2i-associated DKA (SGLT2i-DKA) had a significantly longer time to resolution compared to DKA in patients with T1DM. The median time to resolution of acidosis was 36 h (IQR: 24–72) in the SGLT2i-DKA groups versus 18 h (IQR: 12–27) for the T1DM-DKA group (p < 0.01). The delay was attributed to the lower total insulin dose administered in the first 24 h (median: 44 vs. 87 units; p = 0.01). The relative euglycemia observed in SGLT2i-DKA may result in undertreatment when standard DKA protocols are applied, highlighting the need for tailored adjustments in glucose and insulin infusions strategies [36].

## Postoperative reintroduction of SGLT2i

Reintroduction of SGLT2i may be considered once the patient has resumed adequate oral intake and other risk factors for ketoacidosis—such as infection, hemodynamic instability, or reduced insulin administration—have been resolved [37].

**Table:** 

Important note 5: Emergency surgical procedures in patients using SGLT2i	
In emergency surgical procedures, SGLT2 inhibitors should be discontinued immediately, and capillary ketone levels should be monitored daily for 3 to 5 days postoperatively, or for as long as the patient remains without adequate oral intake. This approach helps detect and manage euglycemic diabetic ketoacidosis (EDKA) promptly For additional guidance, refer to Important Note 3 (ketonemia and EDKA monitoring) and to the chapters on *Euglycemic diabetic ketoacidosis* and *Hospital hyperglycemia in noncritical patients.* [9, 23]	


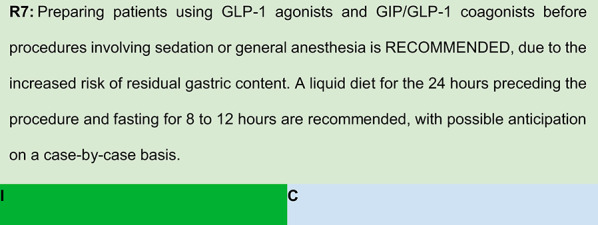


## Summary of evidence

### Impact of diet and preoperative preparation

- Maselli et al. evaluated 57 patients receiving semaglutide, liraglutide, dulaglutide or tirzepatide who underwent endoscopic sleeve gastroplasty without discontinuing their medications. All patients followed a standardized preparation protocol consisting of 24 h without solid foods and 12 h nothing by mouth (*nil per os*, NPO) before the procedure. No residual gastric content was detected during endoscopy, suggesting that this preparation strategy may be a safe alternative to reduce aspiration risk. [38]
- Ghazanfar et al., in a retrospective analysis of 306 patients receiving GLP-1 receptor agonists (GLP-1 RA) undergoing upper GI endoscopy or esophagogastroduodenoscopy (EGD) from 2019 to 2023, compared a clear liquid diet (G1, 41.2%) with a regular diet (G2, 58.8%). Most patients (85.6%) were using GLP-1 RA for diabetes, and 10.1% reported gastrointestinal symptoms beforehand. The incidence of gastric residual content (GRC) was 1.5% in G1 versus 10% in G2 (p = 0.03). Among those with prior digestive symptoms, 13% had GRC, all in the regular-diet group. No procedure-related complications occurred. [39]
- A large systematic review and meta-analysis by Baig et al., including 23 observational studies (262,018 patients), found that GLP-1 RA use was associated with higher odds of GRC (OR = 4.54; 95% CI, 3.30–6.24; p < 0.01) and premature termination of endoscopy procedures (OR = 4.54; 95% CI, 3.05–6.75; p < 0.01). However, there was no increase in aspiration pneumonia. Performing EGD and colonoscopy on the same day was associated with a lower risk of GRC (OR = 0.28; 95% CI, 0.22–0.36; p < 0.01), likely due to the preceding liquid-diet bowel preparation. [40]

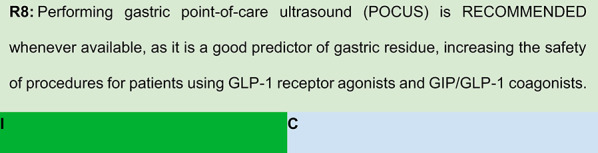


## Summary of evidence

- Kruisselbrink et al., in a controlled study of 40 healthy volunteers, evaluated the accuracy of gastric point-of-care ultrasound (POCUS) after participants underwent an 8-h fast and were then randomized to receive a standard liquid meal, a solid meal, or remain fasted. Each volunteer completed two sessions at least 24 h apart, resulting in 80 total study sessions. POCUS was performed by a blinded sonographer using a standardized protocol. The method demonstrated excellent diagnostic performance, with a sensitivity of 100% (95% CI, 92.5–100%), specificity of 97.5% (95% CI, 95–100%), positive predictive value of 97.6% (95% CI, 87.8–100%), and negative predictive value of 100% (95% CI, 92–100%) [41].
- Nersessian et al., in a prospective study, assessed gastric ultrasound findings in 107 patients who had taken semaglutide within the previous 10 days and 113 control subjects with no recent exposure. Participants were monitored for 2–3 h to evaluate the presence of significant GRC, defined as solid contents or > 1.5 mL/kg of liquid. GRC was observed in 40% of semaglutide users compared with 3% of controls (p < 0.01). In weighted multivariate analysis, semaglutide use (OR 36.97; 95% CI 16.54–99.32; p < 0.01), male sex (OR 2.28; 95% CI 1.29–4.06; p < 0.01), and younger age (OR 0.95 per year; 95% CI 0.93–0.98; p < 0.01) were independently associated with increased GRC. No cases of pulmonary aspiration were reported [42].

**Table:** 

Important note 6: Assessment of gastric residue before the procedure	
Gastric point-of-care ultrasound (POCUS) is a safe, noninvasive bedside tool that can be used to evaluate gastric residual volume (GRV) before procedures. It is particularly useful in patients receiving GLP-1 receptor agonists or GIP/GLP-1 coagonists, and in individuals at increased risk of gastroparesis. [41–43] Identifying gastric residual content (GRC) on POCUS can directly inform perioperative decision-making, including whether to: ● Proceed with the planned procedure, ● Delay or reschedule it, or ● Perform gastric aspiration before anesthesia induction These decisions depend on the urgency of the intervention, the volume and type of gastric contents identified, and the patient’s overall aspiration risk	


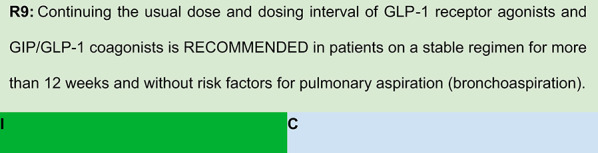


## Summary of evidence

- Singh et al., in a meta-analysis of 23 observational studies including 77,152 endoscopic procedures (4,449 GLP-1 RA users), found that GLP-1 RA use was associated with significantly higher rates of GRC (OR 15.39; 95% CI 4.65–50.99; p < 0.01) and aborted procedures due to gastric residue (OR 13.86; 95% CI 4.42–43.43; p < 0.01). Despite these findings, no significant increase in pulmonary aspiration was observed (OR 21.06; 95% CI 0.13–3,379.01; p = 0.24), indicating that while gastric retention is more frequent, aspiration events remain rare. GLP-1 RA use did not adversely affect bowel preparation quality for colonoscopy (OR 0.94; 95% CI 0.67–1.31; p = 0.83). [44]
- Facciorusso et al. performed a meta-analysis of 13 observational studies (84,065 patients) assessing GRC, aborted endoscopic procedures, and adverse outcomes in GLP-1 RA users. GLP-1 RA therapy was associated with a higher risk of GRC (OR 5.56; 95% CI, 3.35–9.23; I^2^= 72%), which persisted even after adjustment for sex, age, BMI, diabetes, and concomitant therapies (adjusted OR 4.20; 95% CI, 3.42–5.15; I^2^ = 0%). The association was stronger in patients with diabetes (OR 2.60; 95% CI, 2.23–3.02; I^2^ = 24%), suggesting an interaction between diabetes and delayed gastric emptying. GLP-1 RA users also had higher rates of aborted procedures (OR 5.13; 95% CI, 3.01–8.75; I^2^ = 0%) and repeat endoscopies (OR 2.19; 95% CI, 1.43–3.35; I^2^ = 0%). Despite these findings, no significant difference in pulmonary aspiration was observed (OR 1.75; 95% CI, 0.64–4.77; I^2^ = 61%). Subgroup analyses showed that the risk of GRC remained elevated regardless of fasting duration (≥ 12 h, OR 5.47; < 12 h, OR 4.07) with no significant heterogeneity. [45]
- Chen et al., analyzing 366,476 surgical procedures (2020–2022) before ASA’s one-week discontinuation guidance, identified 5,931 GLP-1 RA users (1.6%). After adjustment for demographics, comorbidities, and procedural urgency, GLP-1 RA use was not associated with increased postoperative pneumonia (OR 0.78; 95% CI, 0.57–1.06; p = 0.12) or acute respiratory failure (OR 0.89; p = 0.57). Five sensitivity analyses confirmed these findings. [46]
- Aschen et al. evaluated 74,425 surgical procedures in 21,772 patients, of whom 20,253 were GLP-1 RA users. GLP-1 RA therapy was associated with lower risk of wound dehiscence and hematoma, without increased rates of infection or bleeding. [47]
- Klonoff et al., in a retrospective study of 13,361 adults (2,256 GLP-1 RA users; 11,405 nonusers), found that GLP-1 RA users had lower perioperative complication rates, less delayed gastric emptying, and reduced need for antiemetic medications. No differences were observed in paralytic ileus, pulmonary aspiration, pneumonitis, hypoglycemia, or 30-day mortality. [48]

**Table:** 

Important note 7: Risk factors for gastroparesis	
● Although the use of GLP-1 receptor agonists and GIP/GLP-1 coagonists may increase the likelihood of gastric retention—and potentially the risk of pulmonary aspiration—observational studies have not demonstrated a definitive causal relationship. Several additional factors are known to increase the likelihood of gastroparesis, including poor glycemic control (with risk rising proportionally to HbA1c) and established diabetic gastroparesis, particularly in individuals with more than eight years of diabetes duration. [49, 50] ● Other risk factors include: ● Use of GLP-1 receptor agonists or GIP/GLP-1 coagonists for <12 weeks before the procedure. [51] ● Pre-existing gastroparesis or other gastrointestinal disorders [52] ● Diabetes, especially when accompanied by complications or obesity [52, 53] ● History of pulmonary aspiration or neuromuscular conditions that impair gastric motility [54] ● Presence of gastrointestinal symptoms such as nausea, bloating, early satiety, or abdominal distension [55, 56] ● Use of medications that impair gastric motility, including: [57] ○ Chronic opioids ○ Tricyclic antidepressants ○ Anticholinergic agents ○ Proton pump inhibitors (PPIs) ○ Calcium channel blockers ○ Prokinetics (when initiated prior to GLP-1 receptor agonists therapy)	


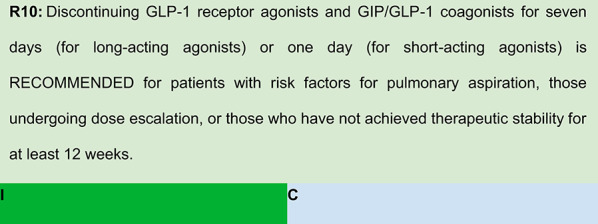


## Summary of evidence

- Phan et al. evaluated 815 GLP-1 RA users undergoing upper endoscopy. Significant gastric retention was observed in 8.7% of patients, and 93% of these cases occurred in individuals with diabetes. After the ASA recommendation to discontinue long-acting GLP-1 RA 7 days before the procedure, the prevalence of gastric retention decreased from 12.7% to 4.4%. However, this reduction did not translate into a significant decrease in the need for orotracheal intubation due to gastric residue (28% vs. 18%). In multivariate analysis, the risk of gastric retention increased proportionally to blood glucose level, rising 36% for each 1% increase in HbA1c, independent of medication type or withdrawal duration. [49]
- Santos et al., in a retrospective study of 1,094 patients undergoing upper endoscopy under sedation or general anesthesia, compared 123 semaglutide users with 971 non-users. GRC was significantly more frequent in semaglutide users (20.3% vs. 3.2%; p < 0.01). The strongest predictor of gastric retention was the presence of digestive symptoms (e.g., nausea, vomiting, dyspepsia), with an OR = 15.1 (95% CI, 9.85–23.45; p < 0.01). Discontinuation of semaglutide for fewer than 8 days was associated with an OR = 10.0 (95% CI, 6.67–15.65; p < 0.01), and for 8–14 days with OR = 4.59 (95% CI, 2.91–7.37; p < 0.01). Only patients who discontinued semaglutide for > 14 days and reported no gastrointestinal symptoms showed no increased risk. These findings suggest that the risk of GRC may persist despite drug withdrawal of up to 14 days, and that persistent GI symptoms may remain an independent risk factor until the end of approximately three half-lives of the medication. [55]

**Table:** 

Important note 8: Time required to discontinue GLP-1 receptor agonists and GIP/GLP-1 coagonists	
When discontinuation of GLP-1 receptor agonists (GLP-1 RA) or GIP/GLP-1 coagonists is indicated, it is important to note that no universally established safe withdrawal period exists. Protocols vary among professional societies, and observational studies suggest that gastric retention may persist even after extended periods of drug discontinuation, especially when gastrointestinal symptoms are ongoing The risk of gastroparesis and potential pulmonary aspiration may remain elevated for up to three drug half-lives, even in the absence of recent administration, although no clear increase in documented pulmonary aspiration events or aspiration pneumonia has been demonstrated. Prolonged discontinuation, however, may worsen glycemic control or lead to weight regain. Notably, poor glycemic control itself is a known risk factor for gastric retention and other perioperative complications. [42, 55, 56, 58] Based on available evidence, the following discontinuation intervals are recommended: Withdrawals intervals ● Long-acting agents (GLP-1 RA or GIP/GLP-1 coagonists): withhold for 7 days before the procedure ● Short-acting agents: withhold for one day before the procedure These measures should be combined with: ● A 24-h liquid diet prior to the procedure, ● 8–12 h of fasting, and ● Preoperative gastric POCUS, when available, to assess residual gastric contents Classification of agents Short-acting GLP-1 receptor agonists: ● Lixisenatide ● Liraglutide Long-acting agonists (GLP-1 RA or GIP/GLP-1 coagonists): ● Dulaglutide ● Semaglutide (oral or subcutaneous) ● Tirzepatide Attention—Fixed-dose insulin and GLP-1 combinations: Fixed-dose combinations containing insulin and GLP-1 RA should be managed according to the properties of the GLP-1 component: ● Glargine + Lixisenatide ● Degludec + Liraglutide The insulin component does not mitigate the gastric-emptying effects of the GLP-1 RA Attention—Colonoscopy preparation considerations: If bowel preparation is inadequate and gastric retention is suspected, a withdrawal period corresponding to three drug half-lives may be considered. If GRC persists, withholding for up to five half-lives may be necessary. However, available data suggest that gastroparesis, rather than colonic inertia, is more commonly responsible for inadequate bowel preparation in GLP-1 RA users. [59–62]

**Table:** 

Important note 9: Similarity between GLP-1 receptor agonists and GIP/GLP-1 coagonists in gastric emptying	
Although GIP/GLP-1 coagonists (such as tirzepatide) have dual hormonal activity, current data indicate that their effects on gastric emptying and on the development of tachyphylaxis are similar to those observed with long-acting GLP-1 receptor agonists (e.g., semaglutide and dulaglutide). [63] Observational studies and meta-analyses have shown no significant difference in the incidence of gastroparesis or residual gastric content when comparing long-acting GLP-1 receptor agonists and GIP/GLP-1 coagonists. [40, 44, 45, 49] These findings suggest that perioperative management strategies related to gastric emptying may be applied similarly across both drug classes

**Table:** 

Important note 10: Preoperative management of patients using GLP-1 receptor agonists and GIP/GLP-1 coagonists undergoing sedation or anesthesia	
For patients with T2DM for require discontinuation of GLP-1 receptor agonists or GIP/GLP-1 coagonists for longer than one day (short-acting agents) or seven days (long-acting agents), it is important to recognize that hyperglycemia is associated with worse clinical outcomes—including delayed gastric emptying. Therefore, the attending physician should be involved early in the preoperative period to evaluate the need for adjustments in antidiabetic therapy, following the recommendations in this chapter and in the guideline *Management of antidiabetic therapy in T2DM.* [8] This collaborative approach helps maintain glycemic stability, mitigate perioperative risks, and prevent deterioration of glycemic control during drug withdrawal	

**Fig. 1:**
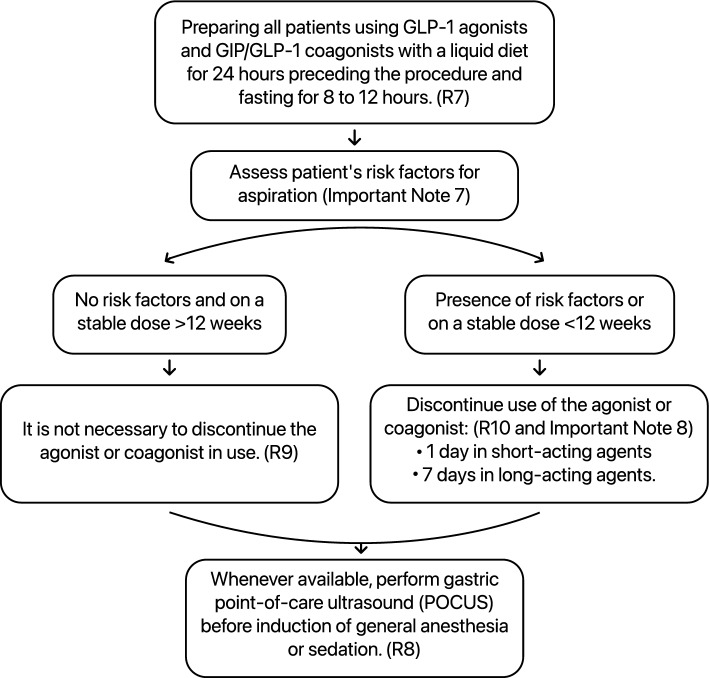
Procedures for patients receiving GLP-1 receptor agonists or GIP/GLP-1 coagonists undergoing sedation or general anesthesia:


To summarize the recommendations and procedures for patients receiving GLP-1 receptor agonists or GIP/GLP-1 coagonists, we provide a flowchart below (Fig. 1):

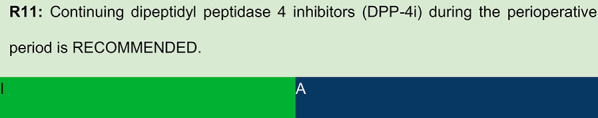


## Summary of evidence

### Safety and efficacy of perioperative use of DPP-4 inhibitors

- Vellanki et al., in a randomized clinical trial of 250 surgical patients, compared linagliptin with a basal-bolus insulin regimen. Among individuals with mild to moderate hyperglycemia (blood glucose < 200 mg/dL), both groups achieved similar glycemic control, but the linagliptin group experienced 86% fewer hypoglycemic episodes. [64]
- Umpierrez et al. evaluated 90 non-critically ill inpatients with good baseline glycemic control, randomized to one of three arms: sitagliptin alone, sitagliptin plus basal insulin, or basal bolus insulin regimen. All groups achieved similar glycemic outcomes, with lower insulin requirements and comparable hypoglycemia rates in the sitagliptin-containing regimens. [65]
- Pasquel et al., in a multicenter randomized trial with 277 medical and surgical patients with blood glucose levels between 140–400 mg/dL, found that sitagliptin plus basal insulin was non-inferior to a basal–bolus insulin regimen in glycemic control and safety. [66]
- Pérez-Belmonte et al. conducted two retrospective studies using propensity score matching to compare DPP-4 inhibitors combined with basal insulin versus basal–bolus insulin in surgical patients. In the first study (227 matched pairs), linagliptin plus basal insulin resulted in glycemic and clinical outcomes comparable to basal–bolus insulin [67]. In the second study (120 matched pairs) including patients with blood glucose levels < 240mg/dL and no previous injectable therapies, the linagliptin + basal insulin group required less insulin and experienced fewer hypoglycemic episodes, with similar glycemic control to basal–bolus regimen. [68]

## Exception

Use of saxagliptin should be avoided, especially in patients with or at risk for heart failure, due to its association with increased hospitalization for heart failure in the SAVOR-TIMI 53 trial [69].

## Use of DPP-4 inhibitors to prevent hyperglycemia in patients without diabetes

Cardona et al., in a randomized clinical trial in 60 patients without diabetes undergoing CABG, evaluated sitagliptin for prevention of stress hyperglycemia. There were no significant differences between sitagliptin and placebo in mean blood glucose levels, incidence of stress hyperglycemia, length of stay, or perioperative complications (reintubation, acute kidney injury, atrial fibrillation). These findings do not support the routine prophylactic use of DPP-4 inhibitors to prevent hyperglycemia in patients without diabetes [70].
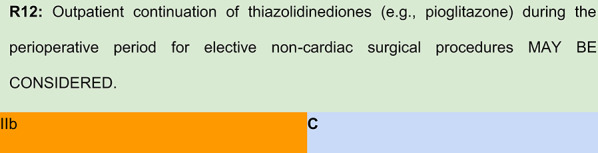


## Summary of evidence

- Preiser et al., in a literature review, suggested that thiazolidinediones (TZDs) may be continued on the day of an elective procedure, provided that the patient is hemodynamically stable and a prolonged hospital stay is not anticipated. [71]
- However, TZDs are associated with fluid retention and may precipitate cardiac decompensation in susceptible individuals. This adverse effect is dose-dependent and cumulative, and its impact may persist for days to weeks even after drug discontinuation. [72]
- The expert panel emphasizes caution when prescribing TZDs in the perioperative setting, especially in patients with known or suspected heart failure, consistent with the recommendations in the chapter *Management of antidiabetic therapy in T2DM.* [8]
- If hospitalization is required, glucose-lowering therapy should follow the guidance outlined in the chapters on *Screening and management of hospital hyperglycemia in non-critical patients.* [9]

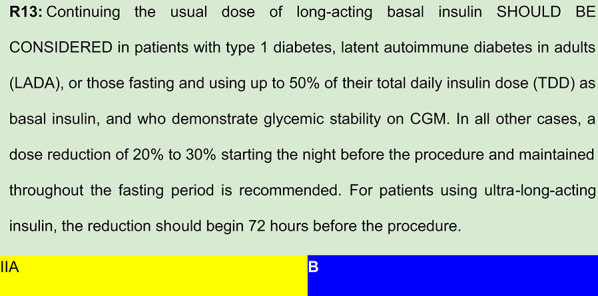


Summary of evidence:

- Demma et al. demonstrated that administering 75% of the usual basal insulin dose starting the night before surgery improved perioperative glycemic control in patients with T2DM. [26]
- Hulst et al., in a retrospective cross-sectional study of 2,259 surgical patients (10% with T1DM, 90% with T2DM), applied a standardized protocol that included discontinuation of oral antidiabetic agents and a 25% reduction in basal insulin the night before surgery. They found that patients with T1DM had significantly worse glycemic control, with higher rates of hyperglycemia (blood glucose ≥ 180 mg/dL; 63% vs. 43%; p < 0.01) and hypoglycemia (7.1% vs. 1.3%; p < 0.01). T1DM patients also exhibited greater glycemic variability and higher HbA1c levels before and after surgery. These findings suggest that a uniform perioperative insulin protocol for both T1DM and T2DM is inadequate, given the inherently greater risk of both hyper- and hypoglycemia in individuals with T1DM. [73]
- The expert panel recommends larger dose reductions in patients using high basal insulin proportions (> 60% of TDD or > 0.6 units/kg/day), with reductions of up to 50% when appropriate.
- For patients using ultra-long-acting basal insulins (e.g. glargine U300, degludec), dose adjustments should begin 48 to 72 h before surgery, due to their prolonged pharmacokinetics profiles. [74]

**Table:** 

Important note 11: Basal insulin in patients with T1DM, LADA, and those who have undergone pancreatectomy	
● Patients with absolute insulin deficiency—including those with type 1 diabetes mellitus (T1DM), latent autoimmune diabetes in adults (LADA), or individuals who have undergone total pancreatectomy—should receive close endocrinology follow-up throughout the perioperative period ● These patients must never have basal insulin omitted, except when receiving: continuous intravenous insulin infusion, or a fully functional subcutaneous insulin pump ● Complete omission of basal insulin may rapidly precipitate diabetic ketoacidosis (DKA) ● As long as the patient is consuming at least 50% of their usual caloric intake, the basal–bolus insulin regimen should be maintained ● When complete fasting, basal–bolus regimen should be transitioned to a basal-plus approach, consisting of: continued basal insulin, and correctional bolus doses of short-, rapid- or ultra-rapid-acting insulin as needed, based on capillary blood glucose levels [75] ● If more than 24 h of basal insulin are missed, capillary ketone testing is recommended. If unavailable, urine ketones should be checked to assess the risk of DKA	

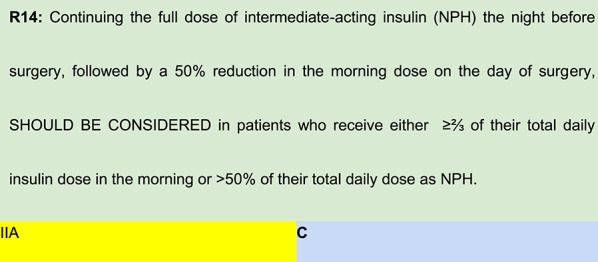



## Summary of evidence

- This recommendation reflects pharmacokinetic differences among insulin formulations and established clinical practice patterns, particularly in settings where intermediate-acting insulin (NPH) is widely used. In many NPH-based regimens, the basal–bolus distribution is frequently unbalanced, with a disproportionately high basal component—often administered in the morning—covering not only fasting but also postprandial periods. Although most supporting evidence comes from studies involving long-acting insulins, adjusting the morning NPH dose on the day of surgery is considered safe—especially for patients who typically receive approximately two-thirds of their total daily dose in the morning. [26, 74]
- In Brazil, many patients on NPH receive a large proportion of their total daily insulin dose in the morning. For these individuals, a 50% reduction of the morning dose is considered safe and may help minimize the risk of hypoglycemia during perioperative fasting.
- For patients already using a NPH-based basal-bolus insulin regimen, maintaining NPH may be reasonable, with proportional dose adjustments (e.g., 50% of each component) and administration of NPH in three equal doses throughout the day. [76, 77]

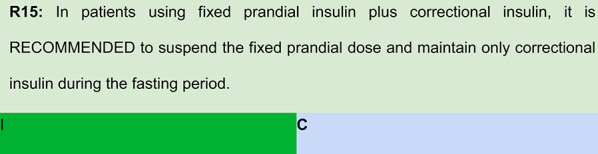


## Summary of evidence

- Fixed prandial insulin doses—whether short-, rapid-, or ultra-rapid-acting—are designed to cover carbohydrate intake and therefore should not be administered when the patient is fasting. During fasting associated with surgical procedures, administering scheduled prandial doses increases the risk of hypoglycemia. [26, 74]
- Patients on a basal-bolus insulin regimen should transition to a basal-plus approach during fasting or periods of dietary interruption. This consists of continuation of basal insulin, use of correctional insulin only for hyperglycemia, and no scheduled prandial doses until oral intakes resumes. [9, 77, 78]
- Further guidance on insulin therapy and dose adjustments can be found in the chapter on *Hospital hyperglycemia in noncritical patients.* [9]

## Summary of recommendations for management of antidiabetic medications

Below, we provide a table summarizing recommendations 4 to 15 (Table 2):

**Table 2:** Preoperative management of antidiabetic medications for elective surgical procedures

Medication	Approach
Biguanide (Metformin)	Hold on the day of the procedure
Thiazolidinedione (Pioglitazone)	May be continued for non-cardiac elective outpatient surgical procedure
GLP-1 receptor agonists Short duration: Lixisenatide and Liraglutide Long duration: Dulaglutide and Semaglutide	1. Liquid diet 24 h prior to procedure, and 8–12 h of fasting (with possible reduction in fasting period); 2. Gastric point-of-care ultrasound (POCUS) to assess gastric residue before inducing anesthesia whenever available; (Important note 10) 3. Discontinuation of medication for seven days (long-acting agonists) or one day (short-acting agonists) for patients with risk factors for pulmonary aspiration, in dose scaling or who have had dose instability in the last 12 weeks. (Important note 8)
GIP/GLP-1 coagonists Long duration: Tirzepatide	1. Liquid diet 24 h prior to procedure, and 8–12 h of fasting (with possible reduction in fasting period); 2. Gastric point-of-care ultrasound (POCUS) to assess gastric residue before inducing anesthesia whenever available; (Important note 10) 3. Discontinuation of medication for seven days for patients with risk factors for pulmonary aspiration, in dose scaling or who have had dose instability in the last 12 weeks. (Important note 8)
DPP-4 inhibitor (Alogliptin, Sitagliptin, Linagliptin, Evogliptin, Vildagliptin, and Saxagliptin)	Continuation is recommended. Saxagliptin should be avoided, particularly in patients with or at risk for heart failure, due to increased risk of hospitalization
SGLT-2 inhibitor (Canagliflozin, Dapagliflozin, Empagliflozin)	Discontinue three to four days before the procedure in cases with caloric intake restriction or major surgical procedures for patients with diabetes. Continue in all other cases
Alpha-glucosidase inhibitor (Acarbose)	Discontinue on the day of procedure
Sulfonylurea (Glibenclamide, Glimepiride, Gliclazide, and Chlorpropamide)	Discontinue 24 h before the procedure
Meglitinide/Glinide (Repaglinide)	Discontinue on the day of the procedure
Prandial insulin (short-, rapid-, or ultra-rapid-acting insulin) (Regular insulin and analogues: Aspart, Lispro, Glulisine, and Aspart with niacinamide)	● Discontinue prandial bolus during fasting period ○ Use only to correct hyperglycemia
Basal insulin (intermediate-acting insulin/NPH)	• Reduce morning dose by 50% for patients: ● Using > 50% of total daily insulin (TDI) as NPH; ● Using ≥ 2/3 of the TDI in the morning • In basal–bolus regimens with proportional doses: ● Maintain NPH with 50% reduction in each dose, administered in three equal daily injections
Basal insulin (long-acting insulin analog: Glargine)	Reducing the dose 20%-30% starting the night before the procedure, with the following precaution: ● In patients receiving a high proportion of basal insulin (> 50% of total daily insulin), consider reducing the dose by up to 50% ● In patients on a basal–bolus regimen with < 50% of total daily insulin as basal, dose reduction may not be necessary. In such cases, maintaining the usual basal dose may minimize hyperglycemia and glycemic variability
Basal insulin (ultra-long-acting insulin analog: Degludec)	Reducing the dose 20%-30%, starting 72 h before the procedure, with the following precaution: ● In patients receiving a high proportion of basal insulin (> 50% of total daily insulin), consider reducing the dose by up to 50% ● In basal–bolus regimens with < 50% of total daily insulin as basal, dose reduction may not be necessary. Maintaining the usual basal dose in such cases may help prevent hyperglycemia and glycemic variability
Continuous interstitial glucose monitors	May remain in place during the procedure, as long as they are outside the surgical field, even when electrocautery is used. Caution is advised when interpreting readings intraoperatively. Interference and inaccuracy may occur; correlate with capillary glucose if needed (Important note 18)
Insulin pumps	May be maintained, as long as the team is familiar with the technology If discontinuing use is required, prescribe effective transition to insulin therapy (basal-bolus, basal with correction, or continuous intravenous insulin) starting 2 h before pump withdrawal to prevent ketoacidosis, which may occur as soon as 90–120 min after discontinuation. (Important note 19)


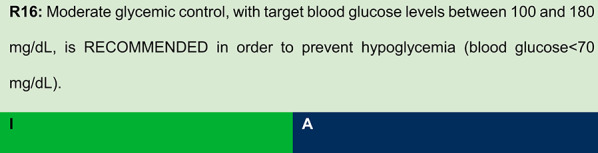


## Summary of evidence

- Umpierrez et al., conducted a randomized controlled trial in patients undergoing CABG, using a computer-guided algorithm to adjust intravenous insulin infusion rates. Among patients without known diabetes, targeting tighter glucose levels (100–140 mg/dL) did not significantly reduce perioperative complications compared with a moderate target (141–180 mg/dL). A subgroup analysis suggested a potential benefit of tighter control specifically in patients without diabetes. [79]
- Sathya et al., in a systematic review and meta-analysis, found that moderate glycemic control was associated with lower postoperative mortality and stroke, with no additional advantage from more intensive control (< 150 mg/dL). [80]
- Abdelmalak et al. noted that tighter glycemic targets may benefit selected patients —particularly those with stress-induced hyperglycemia and no prior diabetes diagnosis. However, they emphasized that such targets carry a higher risk of hypoglycemia, requiring caution application. [81]
- Kalra et al. highlighted that postoperative patients often have blunted counterregulatory responses to hypoglycemia due to sedatives and analgesics, increasing the risk of unrecognized intraoperative and postoperative hypoglycemia. [82]
- Taken together, these data support the recommendations presented in the chapter on *Hyperglycemia in non-critical hospitalized patients*, which also endorses a target range of 100–180 mg/dL for most perioperative scenarios. [9]

**Table:** 

Important note 12: Risk of severe hypoglycemia under general anesthesia	
Hypoglycemia may go unrecognized during general anesthesia, because the typical warning symptoms are absent or masked. If unrecognized and untreated, hypoglycemia can lead to significant perioperative harm, including irreversible neurological injury. Therefore, rigorous intraoperative blood glucose monitoring is essential, particularly in patients receiving insulin or other glucose-lowering therapies. [82]	

**Table:** 

Important note 13: Stricter glycemic targets for individuals without DM	
Evidence suggests that stricter glycemic targets (100–140 mg/dL) may be considered for hospitalized patients with hyperglycemia without a prior diagnosis of diabetes, as this group has been shown to experience higher mortality rates compared with patients who have established diabetes. [6] However, because this approach a higher risk of hypoglycemia—particularly when computerized insulin delivery algorithms are not available—a less intensive target range (100–180 mg/dL) is generally recommended to prioritize safety. [79, 83]	


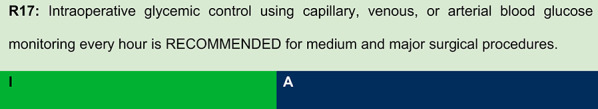


## Summary of evidence

- During the perioperative period of medium and major surgical procedures, hyperglycemia is associated with worse clinical outcomes, including increased morbidity and mortality as well as prolonged hospital stay. [84–86]
- Evidence from a wide range of surgical specialties–including cardiac, thoracic, vascular, orthopedic, neurosurgical, and general surgery– demonstrates that adequate intraoperative glycemic control is associated with improved outcomes, lower complication rates, and faster recovery. [4, 83–89]

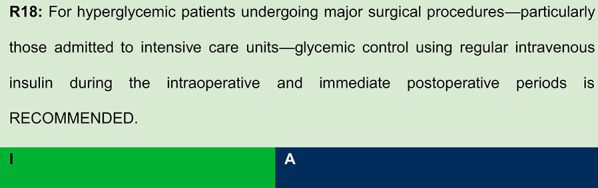


## Summary of evidence

- Intravenous insulin therapy is a safe and effective strategy for glycemic control during the intraoperative and immediately postoperative periods, particularly in critically ill patients [83–89]. Randomized controlled trials in both cardiac and non-cardiac surgeries have demonstrated that appropriate perioperative glycemic control reduces complications and improves clinical outcomes. [4, 83–90]
- The landmark trial by Van den Bergh et al. in critically ill surgical patients (predominantly post–CABG), showed that intensive insulin therapy (target 80–110 mg/dL) with intravenous insulin significantly reduced morbidity and mortality compared with more permissive control (180–200 mg/dL), in a setting characterized by early parenteral nutrition and close monitoring. [84]
- However, a subsequent multicenter randomized clinical trial by the same team, including 9,230 medical and surgical ICU patients treated with computerized insulin algorithms and without early parenteral feeding, found no differences in 90-day mortality or ICU length of stay. These results suggest that the benefits of intensive control may be highly context-dependent. [91]
- Consequently, intravenous insulin infusion remains the standard perioperative strategy for major surgical procedures, but glycemic targets should be more moderate (140–180 mg/dL) to prioritize safety and reduce hypoglycemia risk. [92]
- A 2022 systematic review reinforced that appropriate perioperative glycemic control reduces surgical site infections and other complications. [90]
- Similarly, the NICE-SUGAR trial, which included 38% surgical ICU patients, showed that strict glycemic targets (81–108 mg/dL) were associated with higher rates of severe hypoglycemia and increased 90-day mortality (27.5% vs. 24.9%; OR 1.14; 95% CI 1.01–1.29; p = 0.04), supporting the use of more permissive targets (140-180 mg/dL). [93, 94]
- Because regular insulin has a short half-life (≈5 min), continuous intravenous infusion allows rapid and precise adjustments, helping maintain glucose levels within target range and permitting prompt correction of hypoglycemia when needed.
- Collectively, these findings support the use of regular intravenous insulin during the intra- and immediate postoperative periods for major surgery, with recommended targets of 100–180 mg/dL to prevent hypoglycemia. Infusion rates should be titrated based on hourly blood glucose monitoring.
- Multiple validated protocols are available in the literature, but the safest option is the one that is familiar to and consistently applied by the healthcare team.

**Table:** 

Important note 14: Intravenous insulin protocols	
● Intravenous insulin therapy protocols should include the following core components: ● Hourly blood glucose monitoring to guide safe and timely titration ● Real-time adjustment of infusion rates based on current blood glucose levels ● A target glucose range of 100–180 mg/dL to minimize the risk of hypoglycemia ● Use of a protocol that is familiar to and consistently applied by the healthcare team, as adherence is more important than the choice of any specific algorithm Practical considerations: ● In patients with poor peripheral perfusion, venous or arterial samples are preferred over capillary glucose due to potential inaccuracy ● Many point-of-care glucose meters often have upper limits (typically 500–600 mg/dL); when the device displays “HI,” a venous or arterial sample should be sent for laboratory confirmation ● There is no single ideal insulin infusion rate. Insulin dosing should follow the institutional protocol, with titration aimed at achieving and maintaining glucose within target range. Once glycemic stability is achieved, the infusion rate should be maintained ● The purpose of continuous intravenous insulin infusion is not merely to correct hyperglycemia, but to maintain glycemic homeostasis, analogous to how vasopressors or vasodilators are used to stabilize hemodynamics	

**Table:** 

Important note 15: Dilution, preparation, and substitution of insulin solution	
● Intravenous insulin should be diluted according to the institutional standards, with the most commonly used preparation being 100 units of regular insulin in 100 mL of 0.9% sodium chloride (NaCl 0.9%) ● After dilution and priming of the infusion tubing, it is recommended to discard the first 10% of the solution volume to account for insulin adsorption to the tubing material ● The entire infusion system—including the solution bag, tubing, and connectors—should be replaced at least every 6 h, as progressive insulin adsorption may reduce the effective dose delivered throughout infusion	


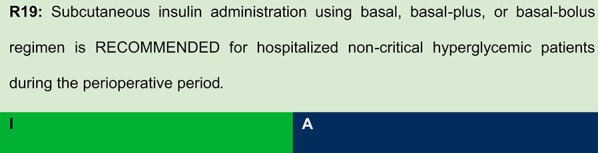


## Summary of evidence

- Subcutaneous insulin regimens—whether basal, basal-plus, or basal–bolus—are considered the gold standard for glycemic control in non-critically ill hospitalized patients. Exclusive reliance on correctional insulin is discouraged because it is associated with poorer glycemic outcomes and higher complication rates. Additional guidance on dose calculation and indications is provided in the chapter on *Hospital hyperglycemia in noncritical patients*. [9]
- Umpierrez et al., in two randomized clinical trials (including RABBIT-2 Surgery), demonstrated that basal-bolus and basal-plus regimens result in better perioperative outcomes than correction-only strategies (sliding-scale insulin). [7, 95]
- In a retrospective analysis of 431,480 surgeries in the Duke University Health System, Van den Boom et al. found that postoperative capillary glucose levels during the first three days were significantly associated with 30-day mortality. In non-cardiac surgery, mortality increased from 1.0% among patients with mean glucose of 100 mg/dL to 1.6% among those with mean glucose of 200 mg/dL. In cardiac procedures, a U-shaped curve was observed: mortality was 4.5% at 100 mg/dL, decreased to 1.5% at 140 mg/dL, and rose again to 6.9% at 200 mg/dL. These findings confirm the risks associated with both hypoglycemia and hyperglycemia during the perioperative period. [2]
- Toyoshima et al., in a retrospective study of 147 predominantly surgical patients managed with the InsulinAPP clinical decision support system, showed that individuals with HbA1c > 8.0% benefited from a basal-bolus insulin regimen, while those with HbA1c below 8.0% achieved adequate control using a simplified prandial-correction regimen consisting of three equal doses of prandial insulin combined with hyperglycemia correction dose (bolus-correction regimen). These findings support the use of personalized insulin strategies based on preoperative glycemic status. [76]
- Collectively, these data confirm that scheduled subcutaneous insulin therapy provides better glycemic stability and superior clinical outcomes compared with correction-only strategies in non-critical perioperative care.

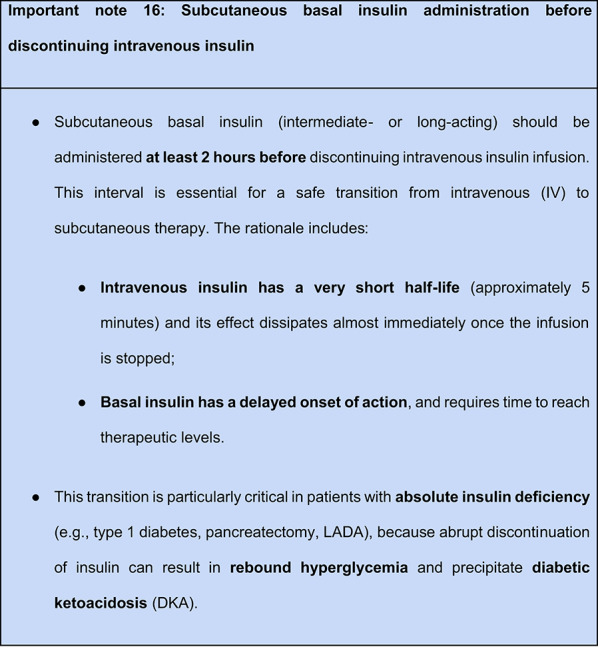
 
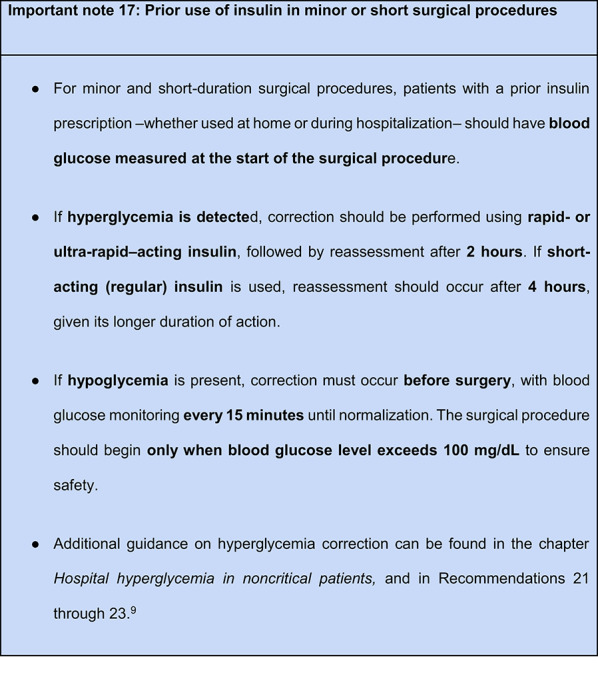
 
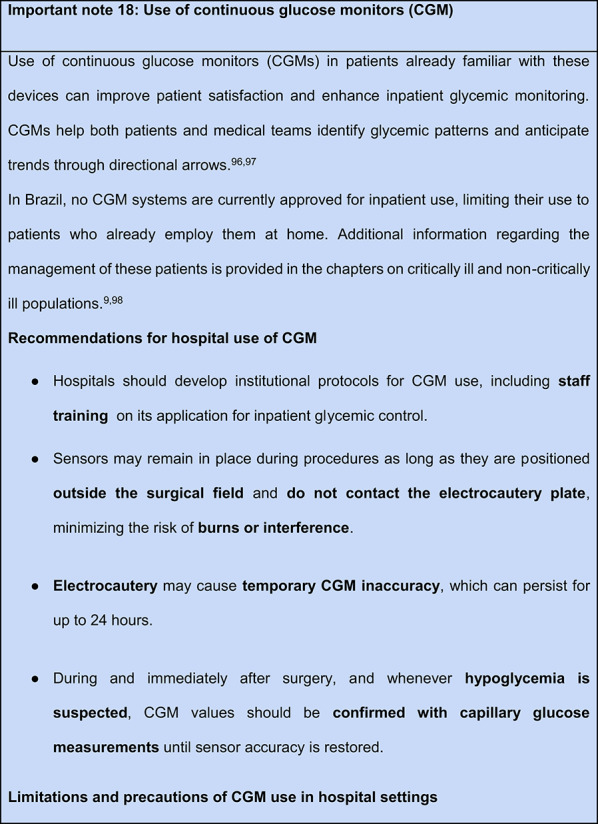
 
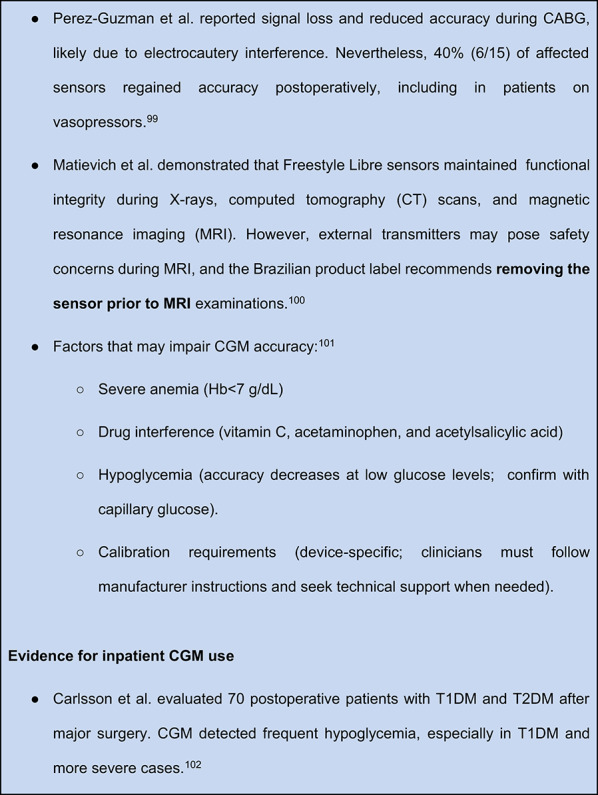
 
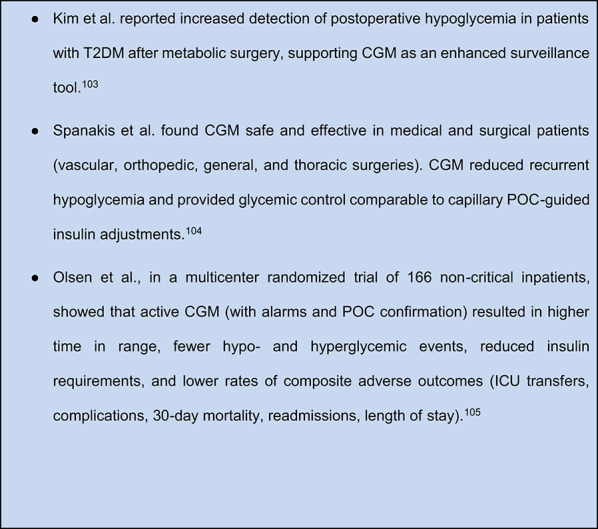
 
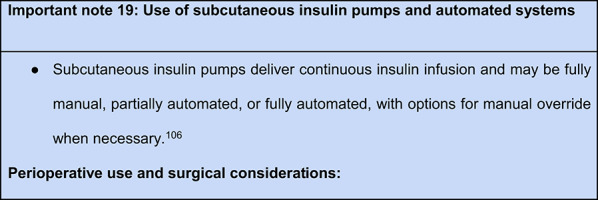
 
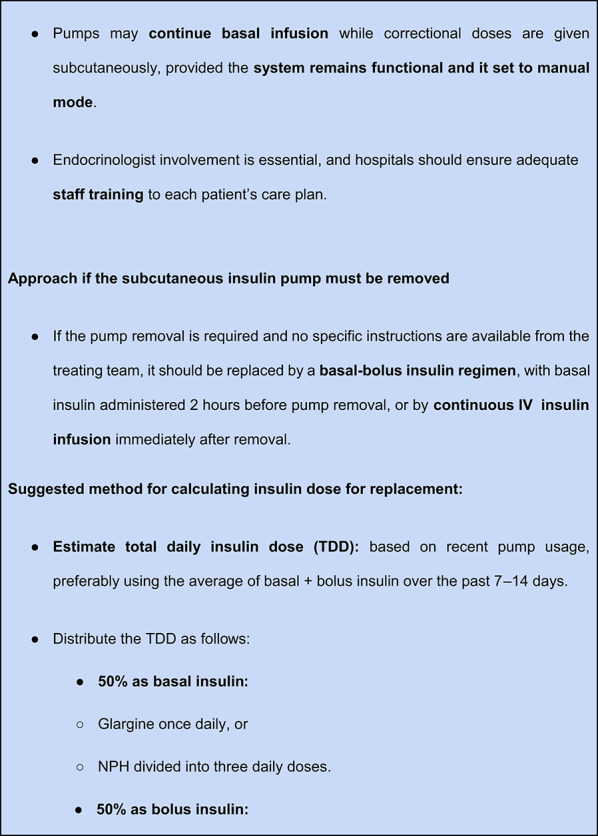
 
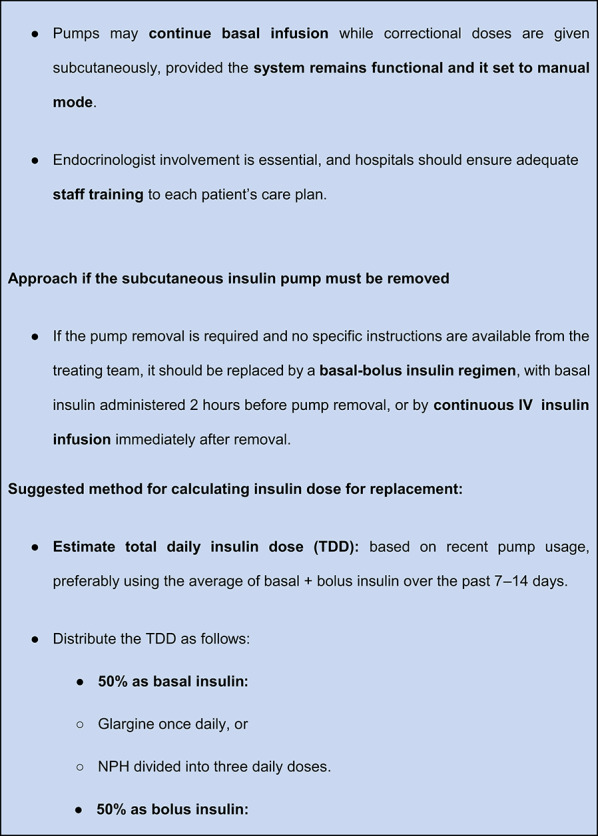
 
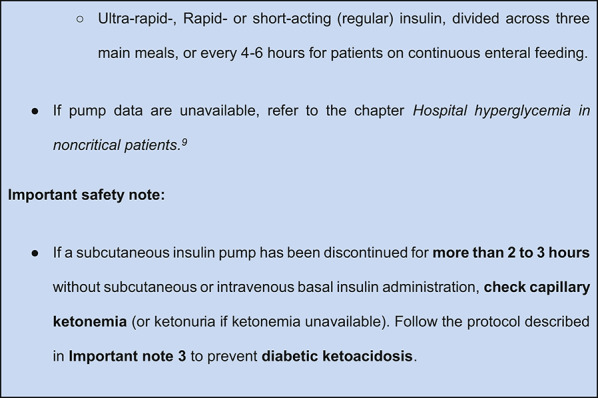
 
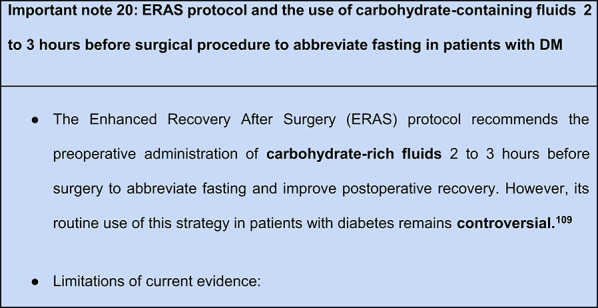
 
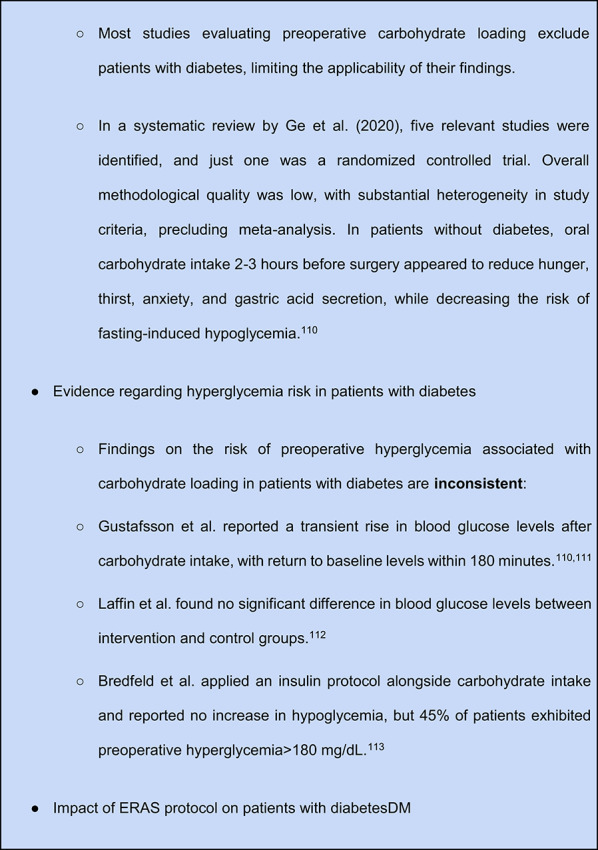
 
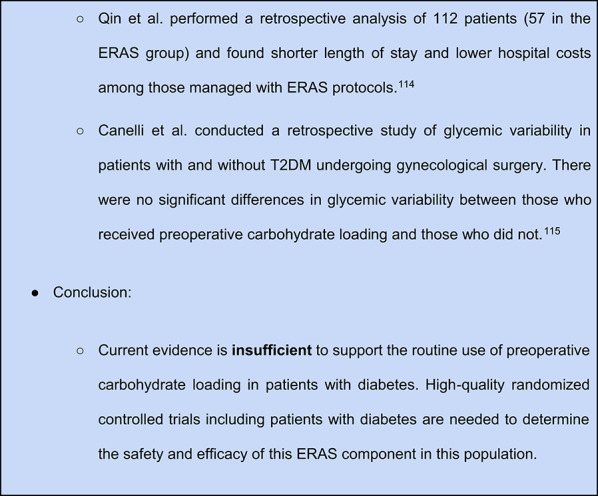


## Table of recommendations


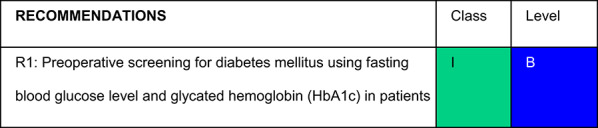
 
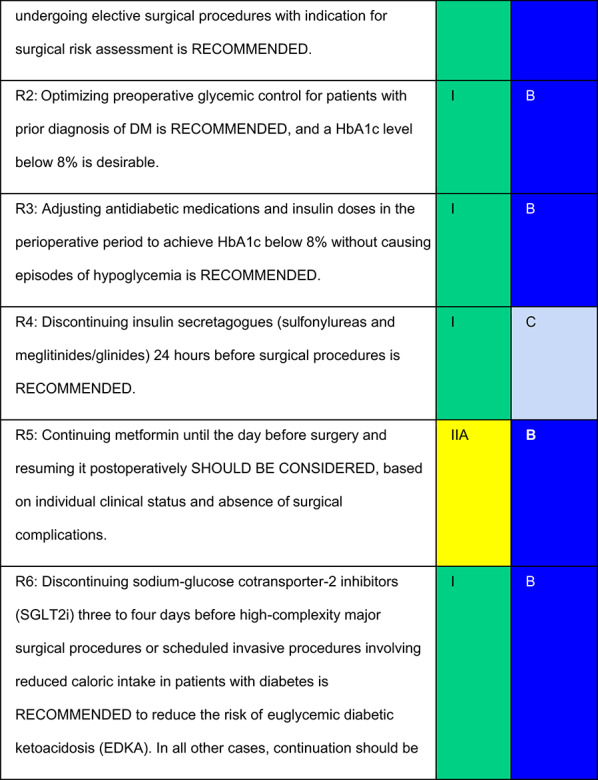
 
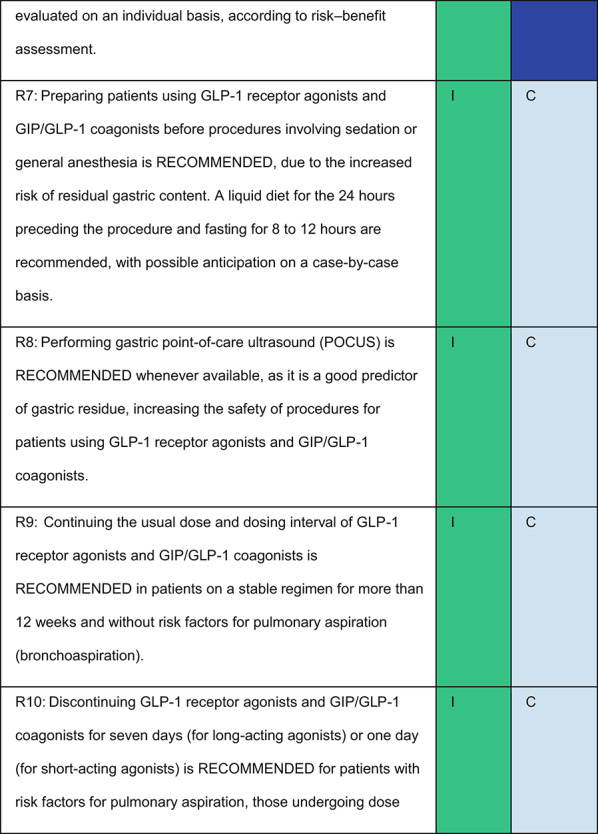
 
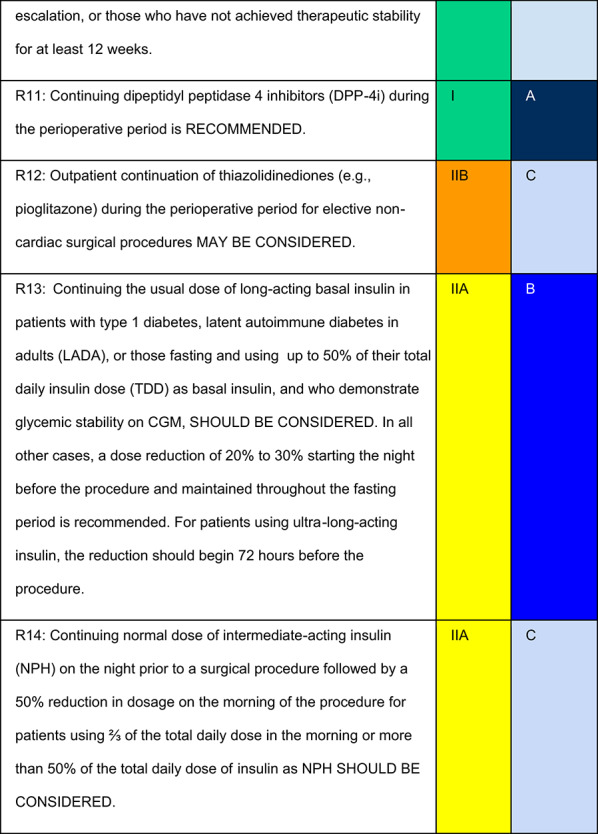
 
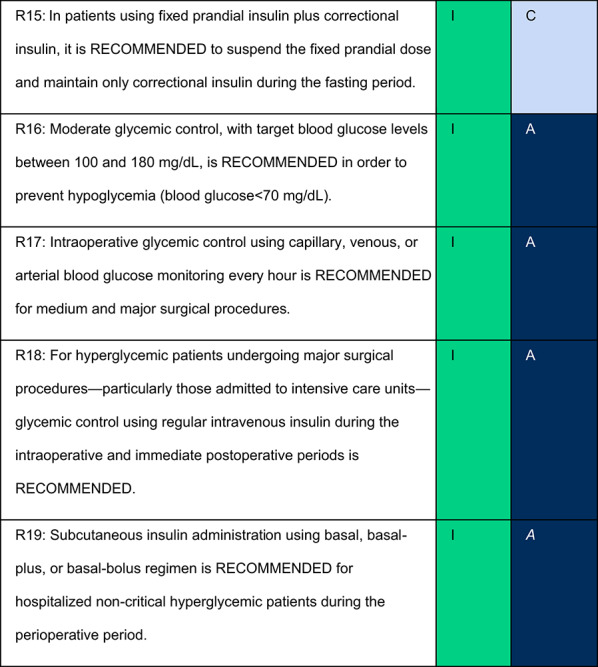


## Data Availability

No datasets were generated or analysed during the current study.
